# Government Commissions of Inquiry and Moralities in Swedish Drug Policymaking

**DOI:** 10.1177/14550725261462373

**Published:** 2026-07-28

**Authors:** Tuulia Lerkkanen, Jessica Storbjörk, Lena Eriksson

**Affiliations:** 1 Centre for Social Research on Alcohol and Drugs (SoRAD), Department of Public Health Sciences, 7675 Stockholm University, Stockholm, Sweden

**Keywords:** drug policy, stakeholders, morality, harm reduction, Sweden, commissions of inquiry

## Abstract

**Aims:**

Government commissions of inquiry (CI) have a long-standing role in policymaking in Sweden, alongside structured consultation processes with stakeholders. This study analyzes stakeholders’ perceptions of CI in Swedish drug policymaking and their moralities regarding a key proposal in the most recent CI: replacing the policy aim “drug-free society” with “a society with reduced harm from drugs”.

**Methods:**

Semi-structured interviews with national-level stakeholders (*N* = 38) and stakeholders’ written comments to the CI (*N* = 76) were analyzed. The Policy Constellations (PC) approach was applied to identify moralities.

**Results:**

Although stakeholders value CI, their role in drug policymaking appeared limited. A lack of implementation of recommendations was a shared concern across PCs. Analyzing the drug policy aim revealed three PCs: one favoring the current aim, grounded in conservatism and public health utilitarianism; one supporting revision, motivated by social justice and equity-based public health; and one drawing on both equity-based public health and public health utilitarianism, supporting modifying the aim in alternate ways.

**Conclusions:**

Stakeholders’ perceptions underscore the need to scrutinize and strengthen CI's role in drug policymaking to support effective policy implementation and legitimacy. The significance of CI fractures in practice, as directives and implementation appear largely grounded in moralities, highlighting key moral tensions surrounding the Swedish drug policy aim. The predominance of utilitarian over equity-based public health approach may explain Sweden's cautious development toward harm reduction.

## Introduction

Governing substance use, particularly the formulation and implementation of illicit drug policy, is challenging and often described as a messy policy area. Many countries envision effective and evidence-based policymaking, which clashes with the reality of contested values and moralities, as well as stakeholders with competing perspectives seeking to participate in drug policymaking (Ritter, 2021; Singleton & Rubin, 2014). In the Nordic countries, societal changes connected to drug use are often under scrutiny, and political debates discuss how to tackle increasing drug use (Tham 2021).

This study highlights the significance of a key stage in policy formation: government commissions of inquiry (CI). These advisory bodies established by governments investigate matters of public and political concern. Alongside structured consultation processes with stakeholders (e.g., experts and government agencies), CI are common in several countries (Christensen et al., 2026; Dahlström et al., 2021; Rowe & McAllister, 2006). This study focuses on Sweden, where the government submits virtually all substantial legislative initiatives to CI at the outset of the policy process (Dahlström et al., 2021; Petersson, 2016; Riksdagen, 2025). CI are appointed by the government to investigate a specific task. They also provide policy recommendations, published as commission reports, which are sent out for comments to stakeholders. After stakeholder consultation, the responsible ministry drafts a bill that is then submitted to Parliament.

In this study, we explore CI as part of Swedish drug policymaking from two perspectives: First, we focus on stakeholders’ perceptions of CI. Stakeholders engaged in agenda-setting, knowledge production, and implementation play a key role in drug policymaking, not least in their role as recurring advisory bodies to CI (Frank et al., 2015; Houborg et al., 2016). We define stakeholders as policy actors “not only as interest groups but also as active or passive players on the policy scene who are also affected by the policy” (Brugha & Varvasovszky, 2000, p. 240). Previous research has examined forms of stakeholder participation in drug policy processes (Duke et al., 2013; Madden, 2022), as well as their strategies, opportunities, and perceptions of power to influence drug policy (Lerkkanen et al., 2026). Furthermore, within substance use research, CI has recently been utilized to analyze, for example, alcohol industry involvement in policymaking (O’Brien et al., 2023) and problem presentations (Kankainen et al., 2024). To our knowledge, no research has examined stakeholders’ perceptions of CI as a tool for drug policymaking. Therefore, by analyzing key informant interviews, this study asks: *How do stakeholders perceive the role of CI in drug policymaking?*

Second, since the Swedish system of CI likely reflects and has shaped how moralities in drug policy debates and agendas develop, we analyze views and comments on a proposal by a recent CI to update the aim of Swedish drug policy. The policy has been based on the aim of a drug-free society since the 1980s. Drug use was criminalized in 1988, followed by ever stricter penalties and control. As a result of increasing mortality rates, the aim was recently combined with the ambition that “no one dies as a result of medicine and drug poisoning” (Skr. 2021/22:213, pp. 65–66). Although the number of drug-related deaths decreased by 30 percent between 2015 and 2023 (Folkhälsomyndigheten, 2025), convictions to narcotics-related crimes have tripled in the last decades, highlighting the challenges the country has faced with drug-related issues (Guttormsson, 2025).

Despite the long tradition of a prohibitionist drug policy, the Drug Commission of Inquiry (DCI) unexpectedly proposed in 2023 revising the aim to a “society with reduced harm from drugs” (SOU 2023:62). This provides a fruitful case for examining Swedish stakeholders’ moralities on the current *and* suggested drug policy aim, as well as the extent to which such an inquiry proposal is viable.

Drug policy goals reflect underlying moralities, illuminating beliefs about standards and what is important in life (Ritter, 2021). Examining moralities behind political arguments helps reveal values that guide policymaking (Stevens et al. 2025a). By applying the Policy Constellations (PC) approach (Stevens, 2024) to key informant interviews conducted before the final report by the DCI, and stakeholders’ written comments to the DCI, we ask: *What kind of policy constellations stakeholders form based on their moralities on the drug policy aim?*

Examining stakeholders’ perceptions of a central drug policy tool provides insights into the strengths and weaknesses of drug policymaking processes. Similarly, an analysis of their moralities on drug policy issues identifies value tensions that must be addressed to foster potential drug policy progression (Askew & Ritter, 2025).

### The Swedish Commission of Inquiry (CI) Context and Drug Commission of Inquiry (DCI)

Sweden can be characterized as a liberal democracy with corporatist traditions (Dahlström et al., 2021). Swedish policymaking has historically been described as consensual, pragmatic and rational, in which the system of CI and a deeply institutionalized faith in science has played a remarkable part (Bergh & Erlingsson, 2025; Dahlström et al., 2021, Eriksson & Edman, 2017). However, previous research has illustrated how internal and external pressures have shaped CI in new directions during the past decades (Bergh & Erlingsson, 2025; Christensen et al., 2026; Dahlström et al., 2021; Petersson, 2016). For example, CI have shifted away from broader commissions with a broad representation of stakeholders and political parties toward a special investigator model, where a single civil servant or judge leads the inquiry, supported by experts, secretaries, and sometimes business or civil society representatives (Dahlström et al., 2021; Petersson, 2016). Interest organizations have experienced a decreasing influence in CI, with CI perceived as “weakened”, partly as a result of shorter time frames and narrower directives (Lundberg, 2015a, 2015b). Sweden's response to the call for more evidence-based policymaking at the expense of politicization has not resulted in a strengthening of academic experts’ position in CI (Christensen et al., 2026). This has been interpreted as an indication of the government's increasing political control, involving the restriction of dissenting voices and the reliance on government-appointed public servants to conduct inquiries.

Research shows that the stakeholder consultation in CI encourages many organizations to participate in policymaking (Lundberg, 2013), although nongovernmental organizations (NGOs) that are already active within the policy field are more often invited. Lerkkanen et al. (2026) further demonstrate that stakeholders within the Swedish drug policy field perceive comments to CI from organizations such as government agencies more influential than those from less established stakeholders. Nevertheless, participation in stakeholder consultation for example by submitting comments to CI may be considered as direct influence strategies. For government agencies, these activities represent a formal institutional duty. Furthermore, their scope is limited, since they are not permitted to “drive politics” or express themselves as freely as some other stakeholders (Lerkkanen et al., 2026).

The most recent drug policy related CI in Sweden is the DCI. In 2020, the Parliament's Committee on Health and Welfare proposed that the Government must evaluate the national drug policy (Riksdagen, 2020). Parliament approved the proposal. In 2022, the Government announced an inquiry which excluded the Penal Law on Narcotics (SFS 1968:64) and thereby the evaluation of criminalization of personal drug use. This decision was widely criticized, as the original proposal called for a complete review. The new government in 2022 kept these restrictions and appointed a special investigator.

In October 2023, the official report of the DCI was published (SOU 2023:62). Strikingly, one of the proposals for the Government was to encourage Parliament to update the drug policy aim from a “drug-free society” to a “society with reduced harm from drugs” – a proposal beyond the stipulated assignment, like another proposal for evaluating drug use criminalization. Since stakeholders submitted their comments by April 2024, there has been no public discussion or communication about the DCI or the next steps.

### Drug Policy Goals Reflecting Values and Moralities

The main goals of international drug policy have focused on reducing drug supply and demand (Ritter et al., 2016). Countries formulate their own drug policy goals, influencing both formal policies and implementation (MacCoun & Reuter, 2001). These goals range from zero tolerance to reducing harm and even legalization (Ritter et al., 2016). Most countries adhere to full prohibition, following the three UN Drug Conventions, adopted also by Sweden (Ritter, 2021). The literature demonstrates that developing harm reduction has often happened within restrictive frameworks (Souleymanov & Allman, 2016). However, recent developments demonstrate how the international consensus on drug policy has been fractured when countries have diverged over specific issues, such as harm reduction, cannabis legalization and death penalties (Stevens et al., 2025b). Furthermore, the prohibitionist consensus has been challenged for its negative impact on health, the environment, and human rights, leading several countries to consider or adopt alternatives (Babor et al., 2018; Csete et al., 2016).

Harm reduction was recently accepted by the United Nation Commission on Narcotic Drugs (Ahmad et al., 2024). It is grounded in social justice and equity, aiming to minimize drug-related harm rather than stigmatize individuals or require drug-free status before support (Harm Reduction International, 2026). In Sweden, a social justice perspective has historically been less prominent than the aim to reduce demand (Goldberg, 2004; Lenke & Olsson, 2002). Harm reduction is not formally recognized as an independent part of Swedish drug policy and therefore competes for resources with control, prevention, and treatment (Tham, 2021).

Hence, drug policy goals reflect underlying values and moralities (Ritter, 2021). The growing interest in studying values and moralities of drug policymaking (Stevens et al., 2025a) may be explained by findings showing how drug policymaking, at the same time as emphasizing evidence-based knowledge, is also shaped by underlying values (Eriksson & Edman, 2017; Lerkkanen & Storbjörk, 2023; Zampini, 2018). Studies on moralities in drug policy have adopted multiple theoretical traditions such as moral philosophy (Askew & Bone, 2019; MacCoun & Reuter, 2001), moral foundations theory (Eriksson et al., 2025), Schwartz's theory of basic values (Askew & Ritter, 2023, 2025), as well as the PC approach (Stevens, 2024) applied in this study.

Research illustrates how moralities in drug policymaking both conflict and overlap, creating multiple rationales for different constellations and interactions (Askew & Bone, 2019; Askew & Ritter, 2023, 2025; Eriksson et al., 2025; Lerkkanen & Storbjörk, 2023; Stevens, 2024). For example, a study of people who use drugs (PWUD) in the UK highlighted a complex interplay of resistance to and support for prohibitionist ideology (Askew & Bone, 2019). Furthermore, attitudes emphasizing health and wellbeing were based on soft paternalism (referring to reducing harm to oneself and others) and appeared in arguments both for and against prohibition. Hence, different values and moralities can be combined and nuance policy preferences (Askew & Ritter 2023). The same value can both support and challenge certain preferences and cluster together with other values, relating to a certain policy preference (Askew & Ritter, 2025; Stevens, 2024). For example, Stevens (2024) identified *compassion* (favoring actions reducing others’ suffering) as a shared value across UK drug policy constellations, but the target of compassion varied between constellations.

As concerns Sweden, a study based on policy documents and interviews with needle and syringe program staff and visitors identified main moral dilemmas concerning PWUD's inclusion versus exclusion; general prevention versus harm reduction; and paternalism versus autonomy (Eriksson et al., 2025). The Swedish drug policy media debate has been found to repeatedly return to the restrictiveness of drug policy, with stakeholders identified as proponents, opponents, or neutral toward the prohibitionist policy – positions based on their moral justifications (Lerkkanen & Storbjörk, 2023).

In this study, we understand moralities as normative principles and preferences, illuminating ideas of what is right and wrong (Ritter, 2021; Stevens, 2024; Stevens et al., 2025a). Such moralities relate to values that illustrate the underlying beliefs that motivate action (Schwartz, 1992). According to the PC approach, stakeholders’ moralities are combined with political ideas to shape which values are prioritized in drug policy (Stevens, 2024). In this study, the approach facilitates a systematic overview of stakeholders’ moralities regarding the existing drug policy aim, and a potential new direction in a traditionally prohibitionist country, at the same time as illustrating how stakeholders form policy constellations in this setting. By offering a deeper insight into the moral rationales behind stakeholders’ political preferences, the study also helps us understand the role CI might play in drug policymaking.

## Methods

### Key Informant Interviews and Stakeholders’ Comments

The analysis was based on two datasets: semi-structured interviews with national-level stakeholders (*N* = 38) (the first investigator (TL) conducted 38 interviews including 39 informants. Participants could bring a colleague to the interview if requested, as was the case in one interview) and stakeholders’ comments to the DCI's report (*N* = 76) (Table 1). Guided by findings by Lerkkanen and Storbjörk (2023), interview sampling aimed to cover proponents, opponents, and neutrals of the drug policy's prohibitionist features (Table 1). We identified potential candidates using media data (2015–2021), governmental reports (2018–2021), and policy documents (2018–2023). Snowball sampling was also used for recruiting PWUD and their significant others. Interviews were conducted between April 2022 and July 2023 by the first investigator (TL) in person (*N* = 23), by phone (*N* = 4) or via Zoom (*N* = 11), lasting around 60 min each.

**Table 1.** Sample Characteristics.

Stakeholder categories	Interviews	Written comments included in the analysis	Written comments not commenting on the drug policy aim
Unorganized recreational PWUD	2		
NGOs representing PWUD and/or opposing a restrictive drug policy, including former PWUD and significant others ^a^	5	3	
NGOs promoting restrictive drug policy, including also former PWUD and significant others	3	6	
Other NGOs and associations (varying missions, forms of funding, and links to the political administration)			6
Molders of public opinion ^b^	3		
Professionals: Healthcare and social services ^c^	4		
Researchers ^d^	3		
The judicial system (police, customs, lawyers, and prosecutors)	5		15
Politically governed organizations (including government agencies) involved with health and social issues	4	9	4
Other government agencies including ombudsmen and supervising agencies			17
Top-level policymaking (MPs, people with experiences of ministries)	9		
Municipalities ^c^		21	15
Counties ^c^		8	7
County administrative boards		19	2
Universities ^d^		6	3
Trade unions, professional organizations, and trade/industry organizations		3	6
Other national councils		1	2
**Total**	**38**	**76**	**77**

NGO: nongovernmental organization; MPs: Members of Parliament; PWUD: people who use drugs.

a NGOs representing PWUD and opposing restrictive drug policy were merged into one group, as most informants from these groups had a history of drug use. Additionally, three informants from these groups also had experience as significant others to PWUD.

b This group includes former politicians and civil servants who remain active in the Swedish drug policy debate.

c Municipalities and counties are often authored by social service or health care professionals.

d Responses by universities are typically authored by researchers.

Several informants held multiple stakeholder roles, such as work as a drug-related professional and membership in profession-based associations dealing with drug-related topics. The sample included six informants with drug use backgrounds and four with significant others. Informants were instructed to primarily respond based on their organizational role, unless explicitly stating a personal view (Lundberg, 2015a, 2015b).

All interviewed informants provided their verbal and written informed consent. The study obtained ethical approval from the Swedish Ethical Review Authority (no. 2021-07012-01).

Concerning stakeholders’ comments, 207 organizations were invited as consultation bodies, of which 145 responded. Although permitted, no unsolicited comments were submitted. Notably, many invited stakeholders, such as local NGOs representing PWUD, did not submit comments. All comments were reviewed, and 76 of them addressing the drug policy aim were selected for analysis.

Together, these datasets complement each other by informing us on views of CI in drug policymaking more broadly, and in mapping stakeholders’ moralities regarding the drug policy aim. Furthermore, although interviews provided confidential and experiential viewpoints, written comments provided official statements, perhaps adjusted to reflect what is considered politically feasible. Because civil servants are not permitted to engage in politics, informants from politically governed organizations explicitly shared their personal views. This underscores how stakeholders’ perceptions and moralities may vary both between and within groups (Lerkkanen & Storbjörk, 2023). Some informants were represented also in the analyzed comments, either through a submission from their organization or by authoring a response themselves. Eight organizations were represented in both datasets, but, because of ethical concerns, they are not disclosed.

### Analysis

Datasets were coded in NVivo (https://lumivero.com/products/nvivo). For the first part, stakeholders’ views on CI's role in drug policymaking were analyzed. This focused on identifying the main patterns of the interview data in which stakeholders were asked about the DCI but also several informants themselves brought up previous drug policy related CI in the discussion.

For the second part, the PC approach was applied to analyze key informant interviews and stakeholders’ written comments to the DCI. The approach identifies moralities and forms of power at the level of social and cultural structures to explain how policymaking works (Stevens, 2024). Its core argument is that policy is made through interactions within PCs – loosely connected networks of actors sharing normative preferences and policy goals. Reasoning in PCs is motivated by moralities and material interests. PCs both generate and exercise power to affect policy through the actions of their members, who may hold different forms of power.

In this study, the PC approach was used to identify whether stakeholders formed PCs around the drug policy aim (Stevens, 2024). In practice, this meant identifying *moralities* and *policy positions* (i.e., policy preferences for policy outcomes); stakeholders sharing the same policy position and moralities formed a PC. These were identified through reading and coding the data with a focus on policy positions and stakeholders’ argumentation that can both reveal and reinforce the moralities underlying their preferences. For example, one informant noted that the goal was unrealistic and that Swedish drug policy had failed, later stressing that PWUD deserve treatment, not punishment. By emphasizing humanity and treatment, at the same time as underlining health aspects and minimizing drug-related harm, this informant valued social justice, public health, and compassion (defined in the Results).

Interviews were coded for informants’ views on a drug-free society, whereas stakeholders’ written comments were coded for perspectives on the proposed aim revision. The interviews were conducted when the DCI was ongoing. Although the inquiry was not tasked with addressing the “drug-free society” aim, it came up in interviews as the most well-known feature of Swedish drug policy. Some informants raised it spontaneously, others were asked about it through the interview guide (see Lerkkanen 2026, p. 112), and three did not address it. A few only stated briefly that the aim is unrealistic. Therefore, moralities were identified within the broader interview context. Similarly, moralities were not always clear in written comments focusing directly on the aim, requiring thorough reading and coding to identify them elsewhere in the response.

The authors discussed the interpretations of the analysis. The identified moralities were compared with findings from previous research (Los, 2024; Stevens, 2024) and with research on moralities in Swedish drug policy (Eriksson et al., 2025; Lerkkanen & Storbjörk, 2023). Next, the PCs around the drug policy aim were mapped, categorizing stakeholders who shared the same policy positions and moralities together in a PC (although all did not form a PC as a result of the lack of shared moralities) (Table 2; see also the Results).

**Table 2.** Nine Identified Policy Positions.

1. Maintaining the current aim **(*N*** **=** **42)**^a^
2. Criticizing the current aim, approving the proposed aim revision **(*N*** **=** **41)**^b^
3. “The proposal needs to be evaluated further” **(*N*** **=** **7)**^d^
4. Wanting to combine the old aim with the new aim **(*N*** **=** **6)**^a^
5. Not taking stand for or against **(*N*** **=** **4)**^d^
6. Agreeing it is a good idea to update the aim but opposing the proposed aim revision **(*N*** **=** **4)**^c^
7. Indirectly accepting the new aim **(*N*** **=** **4)**^d^
8. The restrictive policy and how we see and treat PWUD is the problem, not the actual drug policy aim **(*N*** **=** **2)**^b^
9. “We can keep the old aim but we can also change it if we want to have a more realistic aim” **(*N*** **=** **1)**^a^

PWUD: people who use drugs.

a Classified as conservative PC in the analysis; ^b^ classified as progressive PC; ^c^ classified as micro-PC; ^d^ not further classified or analyzed.

## Results

### Stakeholders’ Perceptions of CI in Drug Policymaking

Three PC crystallized, as outlined in Table 3. These identified PCs are analytical constructs derived from discursive material, rather than empirically observable real-world groupings. The analysis identified both shared perceptions of CI and variation across PCs. Many informants across PCs expressed positive views of CI because they support the principle of making good, evidence-based drug policy. One informant representing top-level policymaking described recent drug policy-related inquiries as progressive, and another appreciated that politicians receive ready-made proposals for legislative changes through inquiries (*conservative PC*). Several informants, mainly from the *progressive PC*, highlighted the previous Comorbidity Inquiry (SOU 2021:93) as a good example of CI work, noting that the investigator's inclusion of perspectives from PWUD and their significant others was particularly valued. Many emphasized the importance of ensuring that these voices are heard (Lerkkanen et al., 2026), aligning with research showing growing support for the inclusion of PWUD in drug policymaking (Askew & Bone, 2019; Lancaster et al., 2018). Hence, CI may be trusted as important tools for inclusive and evidence-based drug policy (cf. Bergh & Erlingsson, 2025; Christensen et al., 2026; Eriksson & Edman, 2017).

**Table 3.** Policy Constellations Around the Drug Policy Aim.

	Progressive policy constellation	Conservative policy constellation	Micro policy constellation
**Policy position**	Criticizing the current aim, approving the proposed aim revision	Maintaining the current aim	Agreeing it is a good idea to update the aim but opposing the proposed aim revision
**Moralities**	***Social justice*** ***Public health – Equity view:*** focus on equal health and harm reduction measures important to save lives, addiction as a disease ***Compassion*** toward people who use drugs	***Conservatism*** ***Public health – Utilitarian view*:** focus on prevention, harm reduction measures important to save lives Minority: skeptical toward harm reduction ***Compassion*** toward children, young people, significant others ***Child narrative***	***Public health – Utilitarian & Equity view*:** Focus on prevention and harm reduction measures ***Compassion*** toward people who use drugs and children ***Child narrative***
**Sub-groups**	Liberty wing Favoring ***liberty*** (Stevens, 2024) For decriminalization of drug use and/or legalizing drugs	Traditionalist wing ***Security*** ***Child narrative*** Mild conservative wing Recognizing stigma ***Social justice***	
**Stakeholders**	NGOs representing people who used drugs and/or opposing a restrictive drug policy, researchers, universities, members of parliament (MPs), representatives of the judicial system, molders of public opinion, municipalities, professional associations, politically governed organizations, and professionals (social services and health care)	NGOs promoting the restrictive drug policy, MPs, molders of public opinion, counties, municipalities, county administrative boards, politically governed organizations, and representatives of the judicial system	County administrative boards, one government agency, and one local politician representing a municipality

NGO: nongovernmental organization; MPs: Members of Parliament.

However, regarding the DCI, although informants across PCs argued that there was a need to review Swedish drug policy and many therefore were positive about the inquiry, the majority *across* PCs expressed disappointment with the Government's decision not to review the social and health effects of criminalizing drug use. For example, one molder of public opinion argued (IP-g):

They say they’re doing a thorough review and then they exclude an area that the legislation itself is based on, that governs the drug policy. It falls on its own absurdity. They are afraid that [evaluating drug use criminalization] would send the wrong message.

This concern about sending the wrong message was also raised by *the conservative PC* in discussions of updating the drug policy aim (see below). Another informant representing the judiciary argued, instead, that the government is not afraid of reviewing criminalization of drug use, but rather of the possibility that this could open the doors to proposals such as cannabis legalization. Altogether, these perceptions illustrate the earlier notion of intensified governmental political control over inquiries, including their directives (Christensen et al., 2026). Informants noted that including also criminalization in the DCI would have allowed a more comprehensive overview of current drug policy, highlighting what works and what does not. Many emphasized that this does not imply support for the decriminalization or legalization of drug use. As one civil servant explained, reviewing something is about exploring, not endorsing, alternatives. These critical perceptions illustrate the previously made notion that the meaning of CI has changed – from objectively and factually analyzing a problem, discussing risks and opportunities, and ultimately presenting arguments for a way forward, to focusing on how the government's proposal should be implemented (Janson, 2026). Only a minority, mainly Members of Parliament (MPs) representing the *conservative PC*, were positive about the DCI's directive. Previous critiques of weakened CI and increased government control, replacing a corporatist consensus tradition, are therefore also evident in the drug policy area (Christensen et al., 2026; Dahlström et al., 2021; Lundberg, 2015a, 2015b).

Similarly, in written comments, several stakeholders across PCs criticized the exclusion of the Penal Law on Narcotics. For example, Malmö University (progressive PC) argued that this was problematic as criminalization of personal use and possession has never been reviewed, despite its implications for the implementation of harm reduction efforts (e.g., supervized consumption sites). The DCI did, however, propose reviewing the law and penalties for drug offences in terms of their medical and social consequences, as well as whether people with harmful use or dependence receive adequate care (SOU 2023:62, p. 40), a proposal mainly supported by the progressive PC.

Many informants, mainly representing the progressive PC, also criticized other aspects of the DCI, including the overlap with the (then) ongoing Comorbidity Inquiry, its reliance on a single special investigator, and the short one-year timeframe. This criticism aligns with earlier findings on changes of Swedish CI (Petersson, 2016) and interest organizations’ negative experiences (Lundberg, 2015a, 2015b).

Criticism of CI in the drug policy field, consistent with critiques in other research, has affected trust. Several informants (mainly from the progressive PC) had lost faith in CI. They described CI as “a political game that the Government plays” to appease stakeholders by showing that there is an ongoing inquiry on the topic. One example mentioned across PCs was the inquiry “Better interventions for addiction and dependence” (SOU 2011:35), with many unimplemented proposals. They worried that the same would happen with the DCI and the Comorbidity Inquiry, as demonstrated by a health care professional:

We were very positive about that [2011 inquiry]. We got a lot of good suggestions. But then it was put in the drawer. Those proposals did not materialize. [… CI] become a political process that follows completely different rules about what is politically possible and so on. (IP-u)

Across PCs, informants highlighted the importance of implementation following inquiries. They also described inquiries as slow processes, with governments rarely achieving concrete outcomes before the next election. This resonates with interest organization's criticism that CI are “window dressing,” offering symbolic inclusion through stakeholder consultation via written comments rather than real influence on policy outcomes (Lundberg, 2015b).

Across PCs, informants stressed the power of the Government in controlling inquiry content and timelines, and selecting investigators and stakeholders deemed to provide “serious inputs.” For example, one informant (politically governed organization) noted that, although media reported that their organization supported reviewing decriminalization together with other politically governed organizations, the ministers argued that there were no “serious inputs” supporting this matter.

More positive and autonomous aspects were also highlighted in interviews, namely that stakeholders still have opportunity to influence drug policymaking through inquiries. One informant described how inquiries can gradually shape attitudes toward drugs, reflecting both their instrumental role and the influence of individual investigators, a point raised by several informants. They noted that although directives are set by the Government, the investigator decides which stakeholders are invited to the inquiry (e.g., expert groups). As in the Comorbidity Inquiry, the DCI's investigator included PWUD and their significant others, a decision welcomed by several progressive PC stakeholders, who stressed the importance of involving these groups in policymaking.

In summary, stakeholders generally trusted the CI system as an institution, but some dissatisfaction was evident regarding the lack of implementation, overlapping terms of reference, and constraints – and thus limitations – imposed by the government's narrow directives. This dissatisfaction is consistent with previous research on CI. In addition, the analysis below shows that more pronounced dissatisfaction among the progressive PC regarding CI in drug policymaking can be understood in light of the moralities that currently shape drug policy and government action, as reflected in the constellations’ perceptions of the policy aim. Regardless, many still choose to participate in the debate through written comments, as illustrated below.

### The DCI Proposal to Update the Policy Aim: Moralities in the PCs

Nine policy positions on the drug policy aim were identified (Table 2). The most common ones were “Maintaining the current aim” and “Criticizing the current aim/approving the proposed aim revision.” Stakeholders maintaining the current aim shared moralities (discussed below) and thus formed the *conservative PC*. Similarly, stakeholders who criticized the current aim and/or approved the proposed revision shared moralities, forming the *progressive PC*.

Stakeholders who supported combining the current and new aims, and a stakeholder who argued that the current aim could either be maintained or updated to a realistic one (since there was no problem to combine “repression with harm reduction”), shared moralities similar to those of stakeholders favoring maintaining the current aim. These were therefore placed in the conservative PC. Two stakeholders’ policy position entailed arguing that the problem lay in the current restrictive policy rather than its aim; these were placed in the progressive PC because of their corresponding moralities.

We also identified a third constellation, a *micro-PC* (Stevens, 2023), consisting of a minority of stakeholders who favored updating the aim but opposed the proposed revision.

Stakeholders who took no position or called for further evaluation did not form PCs because of the absence of shared moralities. Four stakeholders indirectly supported the new aim in their responses but were excluded from further analysis because of the lack of argumentation.

The analysis thus identified three PCs (Table 3).

### Conservative Policy Constellation

The conservative PC included several stakeholder groups, such as NGOs promoting the restrictive drug policy and county administrative boards (Table 3). Their reluctance to deviate from a drug-free society aim was identified as *conservatism*. Stakeholders emphasized the importance of maintaining the restrictive drug policy and abstinence from illicit drugs (Stevens, 2024). For example:

A reformulation of the national goal from “a society free from drugs” to “a society with reduced harm from drugs” risks changing the message and increasing drug use in Sweden. The original restrictiveness of the goal formulation may have a preventive effect and may reduce the risk of increased acceptance of drug use. (written comment by Kungsbacka municipality)

The quote illustrates how many stakeholders in this PC defended the current aim by arguing it may have a preventive effect. It also demonstrates how conservatism was often combined with other moralities in stakeholders’ argumentation; most stakeholders in the conservative PC based their arguments on *conservatism* and *public health* with a *utilitarian view*, preferring policy choices that maximize the benefit and minimize the harms for the majority of people (MacKay, 2018). For example, in their response, Swedish National Council on Alcohol and Drug Issues (SLAN) wrote:

SLAN wants to emphasize the importance of Sweden maintaining a restrictive drug policy. It should be possible to maintain the vision of a “drug-free society” while strengthening efforts to reduce the harm caused by drug use. We see no contradiction between these two parts: they are interrelated and form a whole. We also believe that maintaining the criminalization of personal drug use is important in preventing more people from starting to use drugs.

This example illustrates the growing acceptance of harm reduction, including among stakeholders who support the aim of a drug-free society. Indeed, most written comments opposing a revision of the aim suggested that it could be maintained alongside a stronger focus on harm reduction. This resonates with previous findings showing that harm reduction can be combined with prohibitionist drug policy, rather than posing a threat to it (Tammi, 2007).

Two sub-groups were identified within the conservative PC: *traditionalists* and *mild conservatives* (Table 3). Traditionalists defended the current aim by emphasizing *security*, defined as safety, harmony, and stability for self or those with whom one identifies (e.g., social order, family security, national security) (Schwartz, 1992). They also emphasized compassion, favoring actions reducing others’ suffering, mainly targeted toward children and significant others (Stevens, 2024). Traditionalists and others in the conservative PC often used a “child narrative” in their argumentation, framing drug policy around protecting children from harm (Stevens, 2024). For example, The Swedish Agency for Family Law and Parental Support wrote:

The restrictive drug policy signals to all parents that it is important to create a good and safe environment for children and young people to grow up in. Society should therefore continue to work toward drug-free environments, especially for children up to the age of 18.

The quote demonstrates how the traditionalist wing often argued that updating the drug policy aim would send the wrong message, which resonates with how “sending a wrong message” can be understood as a modern version of framing drugs as sinful and drug use as a societal threat (Ritter, 2021). Another risk with the new aim mentioned, was that it might focus too much on single drug-using individuals, despite drug-related harm also affecting society and significant others.

The mild conservative wing, instead, supported maintaining the current aim but emphasized reducing stigma toward PWUD, combining conservatism, compassion, and social justice in their argumentation. For example, Swedish Association of Local Authorities and Regions wrote that they recognize “the importance of more clearly emphasizing the equal value and human rights of all individuals in policies and other documents to bring about cultural change and reduce stigmatization toward people who abuse or are dependent on drugs.” However, they were reluctant to abandon the current goal in favor of focusing solely on harm reduction. They proposed the goal “to reduce drug-related harm and access to drugs, with the long-term vision of a drug-free society”. These findings illustrate how the combinations of different values and moralities provide nuances in policy preferences within the same policy constellation (Askew & Ritter 2023), also illuminating how these particular nuances provide opportunities to push the boundaries to update the aim.

In interviews, the conservative PC representatives described the current aim as good and important, explaining that it sets the tone for how drugs are viewed in society. For one informant representing top-level policymaking, the aim was “to keep drugs out of Swedish society” (IP-t) and ensure that as many people as possible never try or use drugs. Like the progressive PC (below), the conservative PC did not perceive the current aim as realistic. Instead, many considered it as an aspiration to reduce drug availability and use. One molder of public opinion noted that the current aim was never intended to be realistic:

If you had asked me that question in the 1980s, […] I would have said something like a society with as little drugs as possible. I would not have said that the goal was zero, because I have never believed that we could eliminate non-medical use of drugs. (IP-e)

This quote also resonates with how the conservative PC often referred to zero visions in other policy areas (e.g., for suicide) when arguing to maintain the current aim. Other visions were also “unlikely to be fully achieved in the near future, if ever. However, they serve as a guide for the choice of measures and legislation”, as written by the County Administrative Board of Dalarna.

### Progressive Policy Constellation

The progressive PC included several types of stakeholders, such as NGOs representing PWUD and/or opposing a restrictive drug policy, and professional associations (Table 3). In interviews, they vehemently criticized the current aim of a drug-free society. In both datasets, the progressive PC argued that the aim results in unwanted consequences for both individuals and society, such as hindering access to treatment. One informant representing social services, argued:

I think you would have to do that [change the aim]. Because we have people that are addicted in this country. The perspective we have today stands a little in the way of that work. (IP-f)

Other consequences mentioned concerned societal costs (particularly police resources for “chasing” PWUD). One MP noted that although Sweden maintains the idea of a drug-free society in theory, in practice Sweden has moved further away from this aim due to increased drug availability: “So perhaps we should consider whether this is the right use of public funds, people's time, and prison capacity, etc., to maintain this policy.” (IP-q)

The majority in this constellation based their arguments on *social justice*, as in emphasizing equal human value and reducing stigma (Stevens, 2024). Two molders of public opinion reflected that the aim itself is not a problem; instead, due to the restrictive drug policy, the problem is in the way PWUD are seen as criminals. Many perceived the current aim as moralizing, reinforcing stigma around drugs and PWUD:

I see that as part of the moralistic idea that you should be ashamed, and you should feel bad and you should not get any help. (Unorganized recreational PWUD, IP-w)

Support for the proposed aim revision was expressed in several ways in written comments. For example, The Swedish Agency for Health and Care Services Analysis wrote:

We agree … on the need for a new direction in drug policy, grounded in the right to the best possible health. We support efforts to reduce stigmatization and discrimination against people with harmful use or addiction and that preventive, health-promoting, and harm-reducing measures should be tailored to individual needs. We believe that reformulating the aim of drug policy and raising the ambition to reduce the number of drug-related deaths is both commendable and appropriate for the drug policy.

This quote highlights the principle of social justice by emphasizing the need to reduce stigma and discrimination toward PWUD. It also reflects valuing public health, understood as improving health and saving lives, supporting prevention and harm reduction, and recognizing addiction as a disease. From a public health perspective, drug use is seen as a problem at both individual and population levels, with harm reduction focused on concrete measures rather than ideology (Johnson et al., 2023). This PC links public health and harm reduction to an *equity view*: improving health for all at the same time as reducing inequalities in society (MacKay, 2018), rather than being value-neutral (Ritter, 2021). The quote also illustrates *compassion*, emphasizing the importance of helping people in need (Stevens, 2024).

Some informants combined their argumentation on social justice with *liberty*, valuing the freedom to use drugs for personal purposes (Stevens, 2024). This liberty wing perceived the drug-free aim as problematic, arguing that drug use decriminalization could reduce stigma by prioritizing treatment over punishment. The liberty moral position was undetectable in written comments, probably because the Penal Law on Narcotics was explicitly excluded from the DCI.

Similar to the conservative PC, many progressive PC stakeholders considered the current aim unrealistic and argued it must be changed if there is a genuine interest in advancing harm reduction. For instance, one informant from the judicial system viewed the aim as conflicting with harm reduction:

There has been a strong focus in Sweden on ensuring that people do not use any illegal drugs at all. […] So, it's difficult to talk about harm reduction because, in my opinion, the concept includes a kind of acceptance that people will use these drugs we’re talking about. (IP-h)

### Micro Policy Constellation

In stakeholders’ written comments, a minority (Table 3) supported updating the aim, but not as proposed by the DCI. They formed a so-called *micro-PC* (Stevens, 2023), seeking to combine the suggested aim with a prevention perspective. For example, County Administrative Board of Västra Götaland stated that the aim should include two ambitions: reducing drug use *and* drug-related harm:

In line with the report, the County Administrative Board believes that there should be no requirement to be drug-free to take part in initiatives aimed at reducing harm caused by drug use. We also emphasize the importance of the drug policy aim continuing to have a clear focus on reducing the prevalence of drugs in society and preventing use, particularly among children and young people.

As shown by the quote, the micro-PC, similar to the conservative PC, emphasized both protecting children from drugs and reducing the requirement of being drug-free to access to measures aimed at reducing drug-related harm. By underscoring both prevention and harm reduction in their argumentation, this position can be interpreted as valuing public heath through a combination of equity and utilitarian perspectives (MacKay, 2018): support for the DCI's proposal to strengthen harm reduction to reduce drug-related harms among PWUD (equity), alongside maintaining a focus on prevention and reducing overall drug use (utilitarian).

That only a minority suggested reconciling these two aims by underlining both public health perspectives was a surprising finding, given that Swedish drug policy is part of the country's broader public health policy, and that many stakeholders expressed an interest in updating the aim without abandoning a restrictive drug policy. The analysis raises questions about the meaning of the drug policy aim: responses across both datasets illuminate a contradiction whereby stakeholders across PCs perceived the current aim as unrealistic, yet those in the conservative PC still sought to maintain it. This suggests that the aim carries symbolic value, reflecting morality (Ritter, 2021).

Furthermore, this second part of the analysis illuminates that consensus around a drug-free society has fractured among stakeholders, resonating with recent findings on the fragmentation of international drug policy (Stevens et al., 2025b). Similarly, the results indicate growing acceptance of harm reduction among stakeholders and that this does not necessarily pose a threat to restrictive drug policy, as reflected by representatives of the conservative PC, aligning with international trends (Souleymanov & Allman, 2016; Tammi, 2007). Identifying PCs reveals the value nuances underlying policy preferences, advancing our understanding of differences both between and within the PCs.

## Discussion

This study examined the role of government commissions of inquiry (CI) in drug policymaking from the perspective of stakeholders in Sweden. We analyzed both stakeholders’ perceptions of CI in general, with special emphasis on the recent DCI (SOU 2023:62), and identified PCs (Stevens, 2024) based on stakeholders’ moralities on the drug policy aim and updated aim proposed by the DCI. Although the study indicates that stakeholders trust and value CI as an institution, their role in drug policymaking appeared limited in practice. The lack of implementation following inquiries was a shared source of frustration across PCs. Particularly, the progressive PC was critical of CI, perceiving them as a government strategy to control policy outcomes and avoid responsibility, echoing previous descriptions of them as “window dressing” (Lundberg, 2013). Although many informants considered the DCI important, expectations regarding its outcomes were low, based on previous experiences with other CI in the drug policy field.

The declining significance of CI within the Swedish corporatist and consensus-based tradition is thus also evident in drug policy. By examining moralities alongside CI, here in relation to the DCI, we gain a better understanding of why the CI system may crack in practice. The current drug policy aim, as well as government action, are grounded in a conservative morality, as identified in this study. The government's increasing control over CI, through narrowly defined directives and a preference for single investigators, means that stakeholders with alternative moralities are less likely to be heard within inquiries, or content with subsequent policy implementation. Proposals are unlikely to be implemented if they are not aligned with governmental moralities, overriding CI in practice. The results show how the progressive PC, grounded in a different morality, is more dissatisfied with a CI system that does not produce the reforms it seeks. Yet the findings also reveal nuances: the majority of stakeholders in this case expressed dissatisfaction with the exclusion of the Penal Law on Narcotics from the inquiry directives. Hence, government control appears particularly strong in drug policy. Nevertheless, many stakeholders choose, or have the resources, to participate in the consultation process, even though opportunities to be heard and exert influence vary (Lerkkanen et al., 2026).

This is evident in the results, which highlight power imbalances within CI work – particularly regarding PWUD, and likely more pronounced in policy areas such as drug policy compared to fields with strong user representation, such as large population groups or corporations. Many (mainly the progressive PC) appreciated recent drug policy-related CI for their broader stakeholder involvement, highlighting the relevance of combining expert knowledge with lived experiences (Lancaster et al., 2017). However, informants noted that special investigators determine who is invited. In addition, resource disparities among stakeholders shape opportunities to influence drug policy (Lerkkanen et al., 2026), potentially explaining why many invited did not submit comments or refrained from commenting on the policy aim (Table 1). Silence may also reflect jurisdictional constraints because some stakeholders are unauthorized to comment beyond their mandate. Moreover, official statements may be shaped by what is considered politically feasible.

The ultimate power and thereby also moralities of the government in drug policymaking is evident in the case of DCI, as certain policy areas cannot even be evaluated despite strong support. The majority *across* PCs stressed, in both interviews and written comments, the need to evaluate Swedish drug policy, expressing disappointment over the Government's decision to exclude the criminalization of drug use from the DCI. Recent research has taken steps toward such evaluation, finding drug use criminalization to be an expensive, ineffective, and harmful response to the drug problem (Estrada et al., 2023; Stenström et al., 2024).

The importance of moralities is also illuminated in stakeholders’ discussions of the Government's unwillingness to evaluate the criminalization of drug use, and the conservative PC's reluctance to revise the policy aim due to concern about sending the wrong message enabling a potential paradigm shift toward the normalization of drug use in Sweden (Ritter, 2021). However, although the conservative PC opposed revising the aim, they did not reject DCI's proposals to strengthen harm reduction. This suggests growing acceptance of harm reduction in Swedish drug policy, consistent with earlier findings (Holeksa & Richert, 2024; Nordgren et al., 2022). Such acceptance may illustrate combined values of conservatism and public health that does not challenge prohibitionist paradigm (Tammi, 2007). Notably, the mild wing of the conservative PC moves closer to the progressive PC, although it continues to emphasize different aspects – drugs as a threat (to children) and stricter enforcement within conservatism, rather than the emphasis on humanity and a reluctance to blame the individual underpinning the progressive PC. On the other hand, it is evident that other policy priorities outweigh harm reduction based on social justice and human rights, diverging from international trends (Ahmad et al., 2024), because, shortly before the DCI's final report, the Government raised penalties for the sale of small amounts of drugs (Prop. 2022/23:53). This dominance of valuing a utilitarian rather than equity-based public health perspective may explain the cautious development of harm reduction measures in Sweden. By making underlying values visible and better understanding the synergies and diversion of moralities, it may be easier to identify how drug policy debates can be progressed (Askew & Ritter, 2023, 2025; Ritter, 2021).

The frustration with CI suggests practical implications, including the need to ensure that participation is genuinely inclusive and capable of addressing power imbalances (Ritter, 2021). Critical stakeholders may regain trust if CI lead to concrete policy implementation reflecting and balancing views of various stakeholders in the consultation process. However, this requires political will from a majority of decision-makers, which the results of this study indicate is lacking in drug policy (Lerkkanen et al., 2026). Stakeholders’ perceptions further illuminate structural problems within the current CI system due to its changed focus from thoroughly analyzing and discussing the problem, risks and policy alternatives before presenting proposals for a way forward, to investigating how to implement the government's already chosen proposal (Janson, 2026). Addressing these challenges would require rethinking and critically examining CI structures that may hinder effective drug policy implementation.

The identified moralities of the progressive and conservative PCs align with justifications of proponents and opponents of the restrictive drug policy in the Swedish media debate (Lerkkanen & Storbjörk, 2023). For example, similar moralities were identified in the argumentation of stakeholders willing to maintain the restrictive drug policy despite that the question was on the policy issue concerned updating the drug policy aim to strengthen harm reduction, rather than adopting policy alternatives (e.g., cannabis legalization) as debated in the media.

The study demonstrates that the debate on the goal of Swedish drug policy centers on a tension between progressive and conservative PCs. Nevertheless, nuances and shifts across the different PCs are detectable, which may potentially open up for new constellations in the future, as suggested by the identified micro-PC, which favored updating the aim but not as proposed by the DCI. Instead, they wanted to integrate a general prevention perspective with harm reduction, drawing on public health *equity* and *utilitarian* views (MacKay, 2018). This moves beyond the progressive and conservative PCs relying on different public health perspectives (Table 3). Besides criticizing the current aim for reinforcing stigma toward PWUD, in the written comments, the progressive PC drew on social justice, compassion, and equity-based public health (MacKay, 2018), as well as liberty (Table 3, Liberty wing) in arguing for a shift to a society with reduced harm from drugs. In contrast, the conservative PC defended the current aim as worth striving for and emphasized risks associated with revising it, grounding their reasoning in conservatism and utilitarian public health (MacKay, 2018). Although the traditionalist wing stressed security and compassion for children and significant others, the mild conservative wing combined conservatism with social justice by emphasizing stigma reduction at the same time as retaining the current aim.

Crucial for policy development appears to be the extent to which it is possible to combine contradictory understandings of public health, as the progressive and conservative PCs reflect competing public health perspectives. The progressive PC emphasized equity, preferring to improve health for all and reduce inequalities, whereas the conservative PC took a utilitarian view, prioritizing policy choices that maximize health benefits and minimize the harms for the majority of people (MacKay, 2018). Only a minority of stakeholders embraced both perspectives. This flexibility of public health understandings regarding drugs, and how they can have varied meanings by context, aligns with previous findings (Mold, 2018). Compassion was a shared morality, but targeted children and significant others in the conservative PC, and PWUD in the progressive PC. These different nuances support prior findings that actors favoring different policy positions may still share certain moral positions (Askew & Bone, 2019; Askew & Ritter, 2025; Stevens, 2024). They also resonate with a moral dilemma illustrating the vague boundary between general prevention to protect the public, particularly youth, through harsh drug laws, and promoting the health and wellbeing of PWUD (Eriksson et al., 2025); the findings of this study highlight this same dilemma by showing these nuances of valuing public health how stakeholders perceived the current aim as protecting the health of the population by prevention whereas the new aim would strengthen the focus on the health of PWUD. Only a minority of stakeholders (the micro-PC) proposed to “solve” this dilemma by suggesting that the aim could combine both prevention and harm reduction perspectives.

Swedish drug policy has often been described as an exceptional case in comparison with other European countries due to its reputation as a democratic welfare state with strong equity ambitions that has retained a restrictive drug policy based on the aim of a drug-free society (Chatwin, 2016; Tham, 2021). Despite this aim, drug availability and use, as well as drug-related crime, have continued to increase in the country, illustrating that the policy is far from achieving its goal (Guttormsson, 2025). This study shows that the moralities identified do not differ substantially from those mapped in the UK, which can also be recognized as a country with a drug policy based on prohibition (Stevens, 2024). The similarity between the UK and Sweden, underscores the notion that moralities explain drug policymaking and outcomes, thereby supporting the importance of studying values and moralities in drug policy (see Stevens et al., 2025a). A relevant next step would be to examine the occurrence of joint action within these PC groups in real life, and whether joining forces in practice also leads to increased power and through what channels.

## Data Availability

Not applicable except stakeholders' written comments to the DCI (in Swedish) analyzed for this paper are public documents published on the website of the Swedish Parliament (Riksdagen). The referral responses are available online (https://www.regeringen.se/remisser/2024/01/remiss-sou-202362-vi-kan-battre-kunskapsbaserad-narkotikapolitik-med-liv-och-halsa-i-fokus).
